# Supplementary material for: Green leaf volatile sensory calcium transduction in Arabidopsis
Source: Nat Commun. 2023 Oct 17;14:6236. doi: 10.1038/s41467-023-41589-9 (PMC10582025; doi:10.1038/s41467-023-41589-9)
Supplement: Supplementary file 1 — Supplementary Information [file 41467_2023_41589_MOESM1_ESM.pdf]

Green leaf volatile sensory calcium transduction in *Arabidopsis*

Aratani et al.

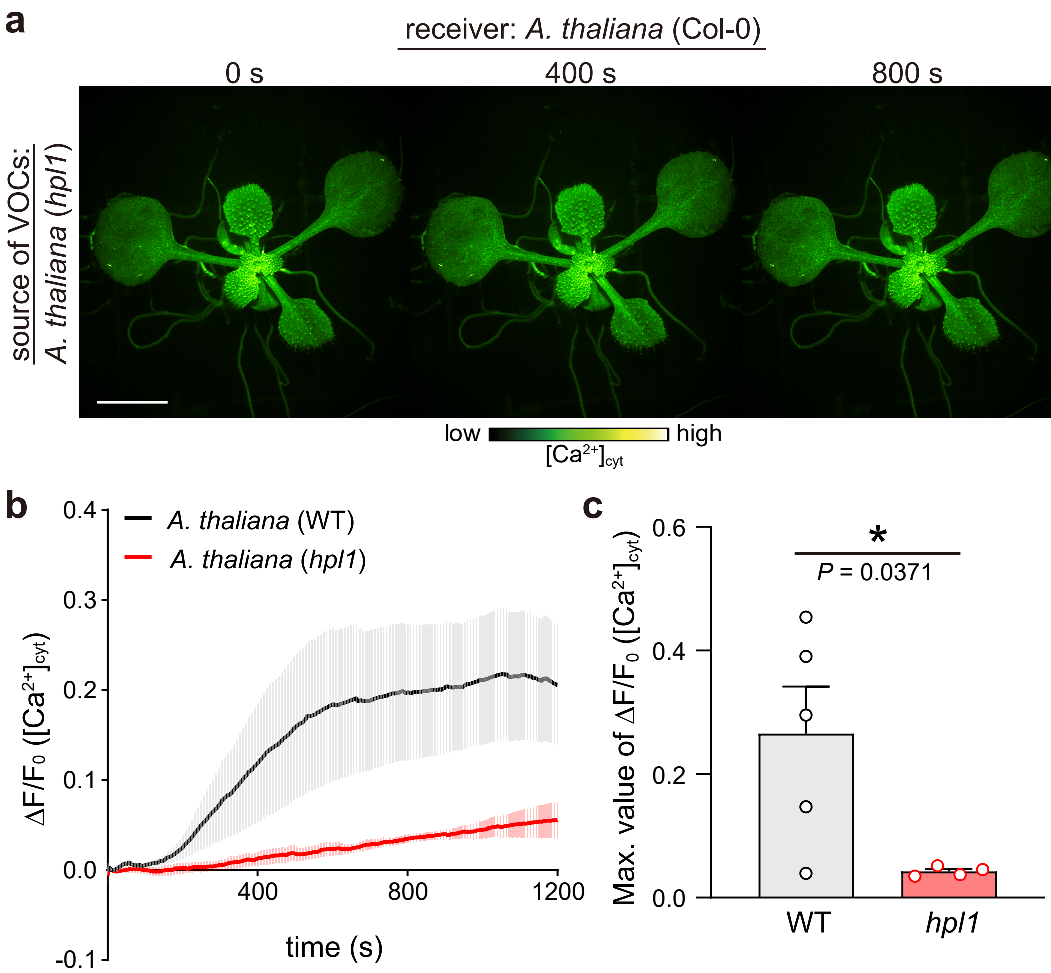

Supplementary Fig. 1. Exposure to VOCs emitted by homogenized *Arabidopsis* leaves carrying inactive *HPL* did not induce $[Ca^{2+}]_{cyt}$ changes in the receiver *Arabidopsis* leaves.

(a) Exposing *Arabidopsis* (receiver) to VOCs emitted from homogenized *Arabidopsis* carrying an inactive *HPL* sequence (*hpl1*) (source of VOCs) did not trigger $[Ca^{2+}]_{cyt}$ increases. Scale bar, 5 mm. **(b)** Quantification of $[Ca^{2+}]_{cyt}$ signatures in leaf 1 (L1) of receiver *Arabidopsis* upon exposure to VOCs emitted by the homogenized tissues of wild-type (WT) *Arabidopsis* and the *hpl1* mutant (*hpl1*). Error bars, mean ± SE. N = 5 and 4 biologically independent samples for *A. thaliana* (WT) and *A. thaliana* (*hpl1*), respectively. **(c)** Comparison of the maximal $[Ca^{2+}]_{cyt}$ changes detected in L1. Error bars, mean ± SE. N = 5 and 4 biologically independent samples for *A. thaliana* (WT) and *A. thaliana* (*hpl1*), respectively. An asterisk denotes a significant difference based on two-tailed Student's *t*-test (*, $P < 0.05$).

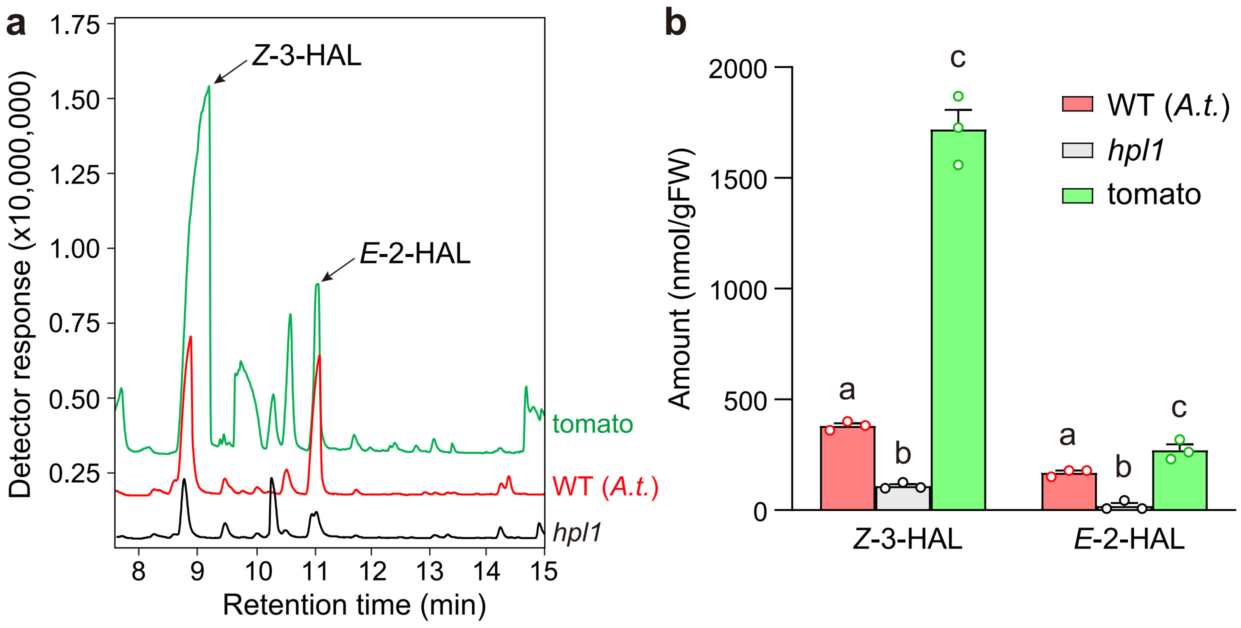

Supplementary Fig. 2. Amounts of volatile compounds formed and emitted by homogenized tomato and *Arabidopsis* tissues.

(a) Total ion chromatogram of VOCs collected from homogenized wild-type *Arabidopsis* [WT (*A.t.*); red], the *hpl1* mutant (black), and tomato plants (green) for 10 min. **(b)** The amounts of *Z*-3-HAL and *E*-2-HAL were quantified from calibration curves constructed with authentic compounds. Error bars, mean ± SE. N = 3 biologically independent samples. Different letters denote significant differences based on one-way ANOVA followed by Tukey's honestly significant difference post hoc test ($P < 0.05$).

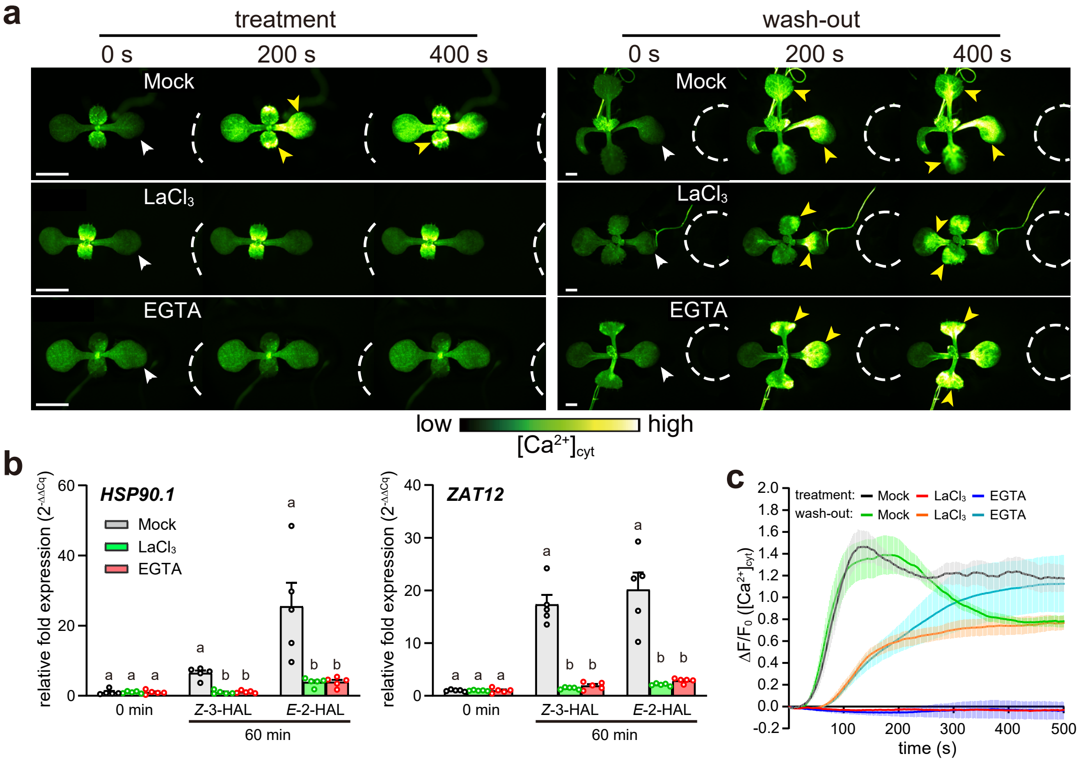

Supplementary Fig. 3. $LaCl_3$ and EGTA block C6 aldehyde-induced $[Ca^{2+}]_{cyt}$ increases and defense gene induction.

(a) Pretreatment of *Arabidopsis* seedlings with the Ca^{2+} channel blocker $LaCl_3$ (50 mM) and Ca^{2+} chelator EGTA (50 mM) inhibited *Z*-3-HAL-induced $[Ca^{2+}]_{cyt}$ increases (yellow arrowheads) (left). After incubation of these seedlings with a medium without the pharmacological reagents for 24 h, the reversibility of Ca^{2+} signals was observed (right). Dashed white lines indicate the position of the plastic tube. Scale bar, 1 mm. **(b)** Defense-related gene (*HSP90.1* and *ZAT12*) induction after exposure to *Z*-3-HAL and *E*-2-HAL. *ACT8* was used as an internal reference for standardization. Error bars, mean ± SE. N = 5 biologically independent samples. Different letters denote significant differences based on one-way ANOVA followed by Bonferroni's post hoc test ($P < 0.05$). **(c)** Quantification of $[Ca^{2+}]_{cyt}$ signatures in the leaf closest to a plastic tube (white arrow). Error bars, mean ± SE. N = 4 biologically independent samples.

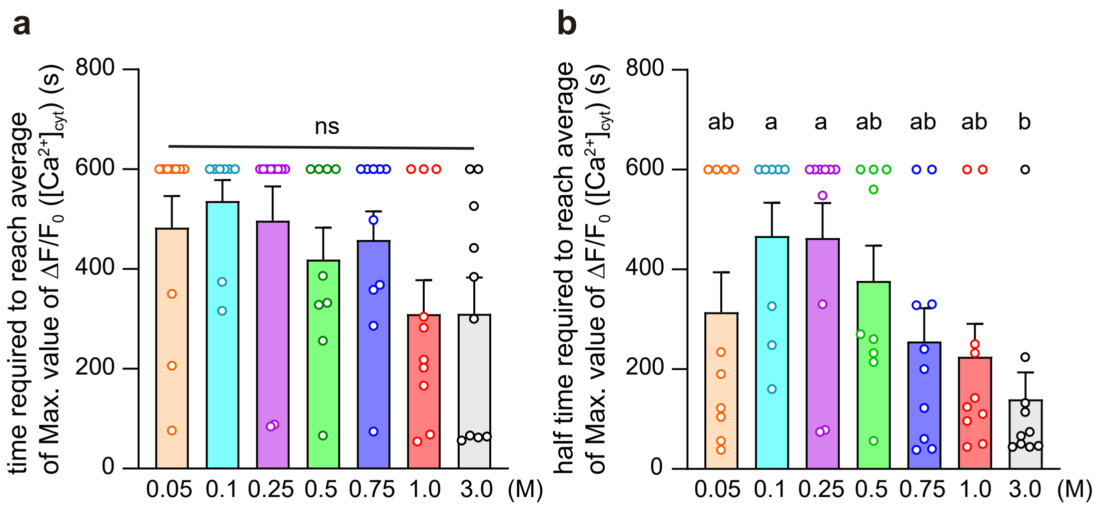

Supplementary Fig. 4. Comparison of the time required to reach the mean value of maximal $[Ca^{2+}]_{cyt}$ changes detected in receiver *Arabidopsis* upon exposure to different concentrations of *Z*-3-HAL.

Although the time required to reach the mean value of maximal $[Ca^{2+}]_{cyt}$ changes upon exposure to different concentrations of *Z*-3-HAL did not exhibit concentration dependency **(a)**, a trend of concentration dependency was observed in the analysis of the half-maximal response (defined as the time required to reach 50% of the mean maximal value) Error bars, mean ± SE. N = 10 biologically independent samples for 0.05, 0.25, 0.75, 1.0 and 3.0 M, N = 8 for 0.1 M, and N = 9 for 0.5 M. ns, not significant. **(b)**. A value of 600 s was assigned to represent those samples that did not reach the mean value. Error bars, mean ± SE. N = 10 biologically independent samples for 0.05, 0.25, 0.75, 1.0 and 3.0 M, N = 8 for 0.1 M, and N = 9 for 0.5 M. Different letters denote statistically significant differences based on one-way ANOVA followed by Tukey's honestly significant difference post hoc test ($P < 0.05$).

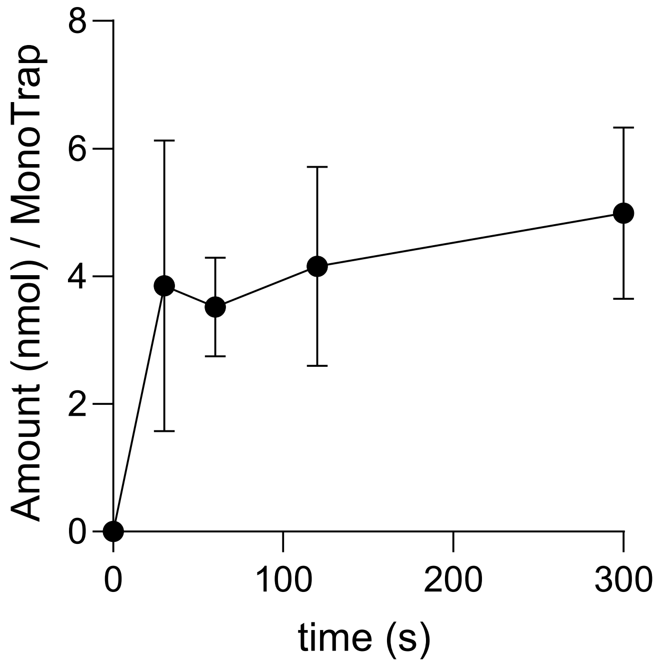

Supplementary Fig. 5. Quantification of *Z*-3-HAL volatilized from *Z*-3-HAL solution.

MonoTrap RGPS TD was positioned at a distance of 5 mm from the DMSO solution containing 0.03 M *Z*-3-HAL, which corresponds to the location of L1 in receiver *Arabidopsis*, for the indicated time points. The amounts of *Z*-3-HAL were quantified by utilizing calibration curves constructed with authentic compounds. Error bars, mean ± SE. N = 3.

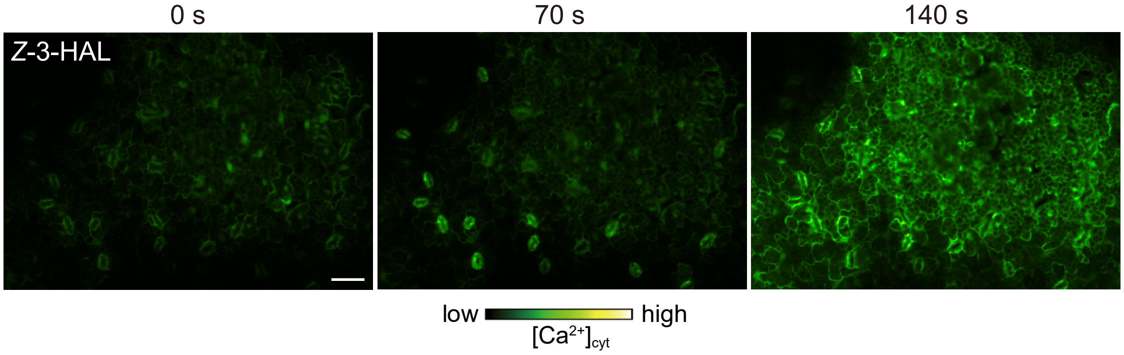

Supplementary Fig. 6. High-resolution images of *Z*-3-HAL-induced $[Ca^{2+}]_{cyt}$ increases obtained using an upright confocal laser scanning microscope.

Time-course changes in $[Ca^{2+}]_{cyt}$ on the surface of *Arabidopsis* leaves expressing GCaMP3 driven under the 35S promoter after exposure to *Z*-3-HAL were observed using a confocal microscope. Upon *Z*-3-HAL exposure, an immediate increase in $[Ca^{2+}]_{cyt}$ was detected in guard cells (70 s). Subsequently, $[Ca^{2+}]_{cyt}$ increases were observed in mesophyll and epidermal cells (140 s). Scale bar, 50 µm.

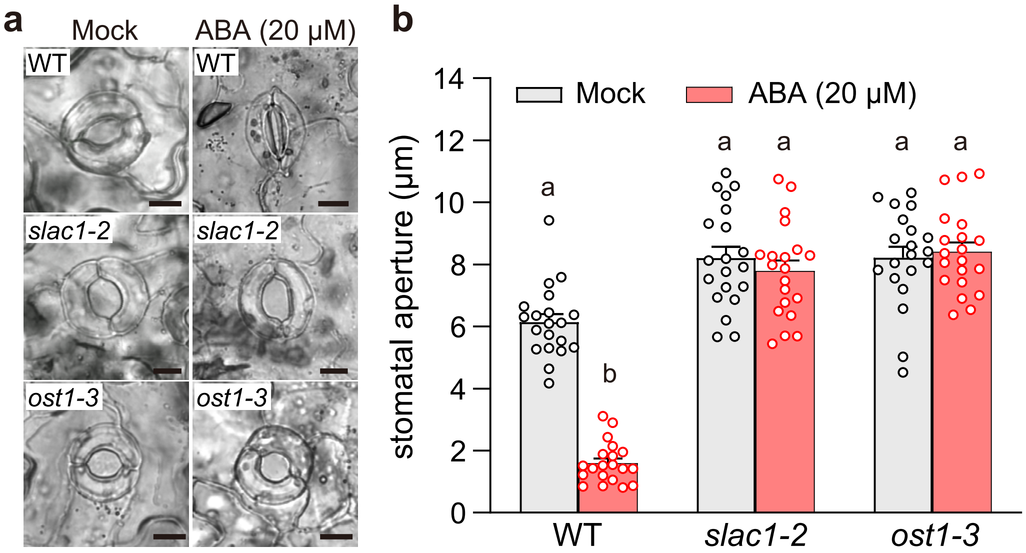

Supplementary Fig. 7. Application of ABA induces stomatal closure in the leaves of wild-type (WT) plants but not in *slac1* and *ost1* mutants.

(a) Images of the stomata in leaves of WT, *slac1-2*, and *ost1-3* plants. Scale bar, 10 μm. **(b)** Stomatal aperture measurements. Detached mature leaves were soaked in opening buffer with or without the application of 20 μM ABA and kept under light. Stomatal apertures were measured 2 h after treatment. Error bars, mean ± SE. N = 20. Different letters denote significant differences based on one-way ANOVA followed by Tukey's honestly significant difference post hoc test ($P < 0.05$).

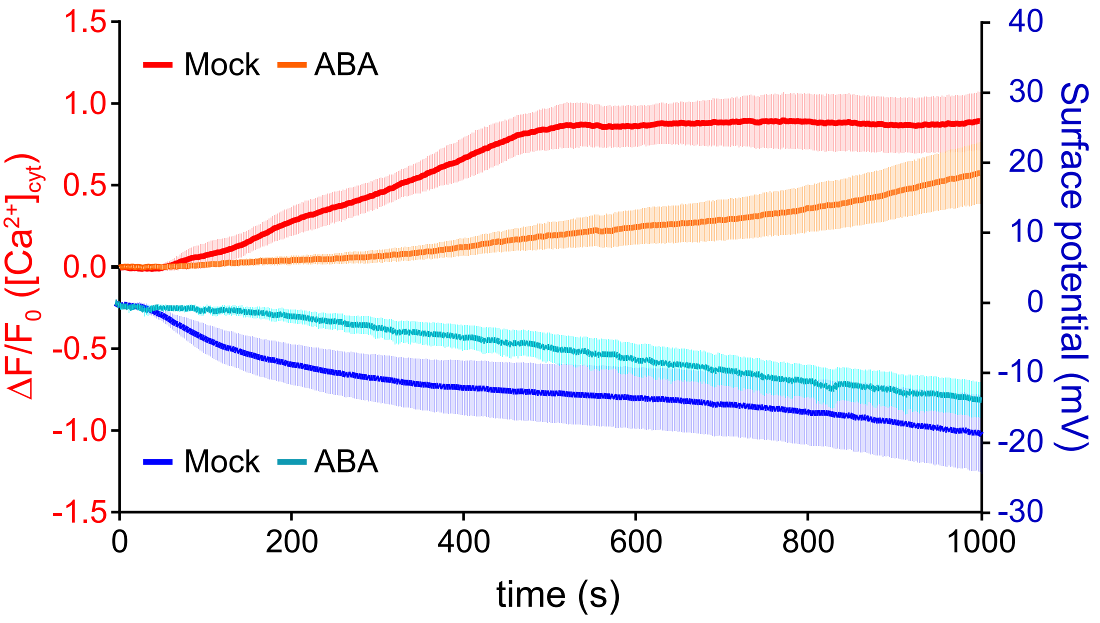

Supplementary Fig. 8. ABA treatment inhibits rapid changes in the leaf surface potential in response to *Z*-3-HAL exposure.

Detached WT leaves were soaked in opening buffer with or without the application of 20 μM ABA and kept under light for 2 h. Simultaneous recording of the changes in the leaf surface potential and $[Ca^{2+}]_{cyt}$ in leaves pretreated with or without ABA was conducted upon the application of *Z*-3-HAL solution. Error bars, mean ± SE. N = 6 biologically independent samples.

Supplementary Table 1. List of primers used in this study.

For cloning

Primer name	5' to 3'
pGC1_F(SphI)	ACATGCATGCGAGTAAAGATTCAGTAACCCG
pGC1(XbaI)_R	CGTCTAGAATTTCTTGAGTAGTGATTTTGAAG
pRBCS1A(SbfI)_F	ACTTCCTGCAGGCTTACGAGGAGCTTGAGCTTCAATG
pRBCS1A(XbaI)_R	CGTCTAGAGTTCTTCTTTACTCTTTGTGTGACTGAGG
pSULTR2;2(HindIII)_F	CGATAAGCTTGACCAAAGAATCCTACGTACC
pSULTR2;2(NheI)_R	CTAGCTAGCGTGGGTTATTGAAGTGTGTGATAGGG
pATML1(HindIII)_F	CGATAAGCTTATCAAAGAAAAAACAAG
pATML1(AscI)_R	TTGGCGCGCCAACCGGTGGATTCAGGGAGT

For qPCR

Primer name	5' to 3'
ACT8-qPCR-Fw	TGCTGGTCGTGACCTTACTG
ACT8-qPCR-Rv	CGAGGTCTCCATCTCTTGCT
OPR3-qPCR-For	CGTTTTACACTCAAGATCCAGTTG
OPR3-qPCR-Rev	ATTATCAAACTCAGAGGCGGG
JAZ7_qPCR-F2	GATCCTCCAACAATCCCAAA
JAZ7_qPCR-R2	TGGTAAGGGGAAGTTGCTTG
HSP90.1-qPCR-Fw	CAGCTTTGTTGACGTCTGGA
HSP90.1-qPCR-Rv	TCGACTTCCTCCATCTTGCT
Zat12-qPCR-F(b)	TCATCAGAAGAAAAATGGTTGCG
Zat12-qPCR-R	AAGCATCAAACAATTCGCCG
