# Supplementary material for: Green leaf volatile sensory calcium transduction in Arabidopsis
Source: Nat Commun. 2023 Oct 17;14:6236. doi: 10.1038/s41467-023-41589-9 (PMC10582025; doi:10.1038/s41467-023-41589-9)
Supplement: Supplementary file 3 — Description of Additional Supplementary Files [file 41467_2023_41589_MOESM3_ESM.pdf]

Description of Additional Supplementary Files

Supplementary Movie 1. $[Ca^{2+}]_{cyt}$ increases in response to VOCs emitted by *Arabidopsis* accession No-0 leaves fed by *S. litura* larvae.

Arabidopsis was exposed to VOCs emitted from No-0 leaves fed by *S.litura*.

Supplementary Movie 2. $[Ca^{2+}]_{cyt}$ increases in response to VOCs emitted by tomato leaves fed by *S. litura* larvae.

Arabidopsis was exposed to VOCs emitted from tomato leaves fed by *S.litura*.

Supplementary Movie 3. $[Ca^{2+}]_{cyt}$ increases in response to VOCs emitted by homogenized *Arabidopsis* accession No-0 leaves.

Arabidopsis was exposed to VOCs emitted from homogenized No-0 leaves.

Supplementary Movie 4. $[Ca^{2+}]_{cyt}$ increases in response to VOCs emitted by homogenized tomato leaves.

Arabidopsis was exposed to VOCs emitted from homogenized tomato leaves.

Supplementary Movie 5. $[Ca^{2+}]_{cyt}$ increases in response to *Z*-3-HAL exposure.

Arabidopsis was exposed to *Z*-3-HAL that volatilized from a solution placed at a distance of 5 mm from the tip region.

Supplementary Movie 6. $[Ca^{2+}]_{cyt}$ increases in response to *E*-2-HAL exposure.

Arabidopsis was exposed to *E*-2-HAL that volatilized from a solution placed at a distance of 5 mm from the tip region.

Supplementary Movie 7. *Z*-3-HAL causes local, but not systemic, $[Ca^{2+}]_{cyt}$ increases in spatially separated *Arabidopsis* leaves.

An *Arabidopsis* leaf that was spatially separated was exposed to *Z*-3-HAL.

Supplementary Movie 8. *Z*-3-HAL–induced $[Ca^{2+}]_{cyt}$ increases in guard cells.

Arabidopsis expressing GCaMP3 under the control of the GC1 promoter was exposed to *Z*-3-HAL that volatilized from a solution placed at a distance of 5 mm from the tip region.

Supplementary Movie 9. *Z*-3-HAL–induced $[Ca^{2+}]_{cyt}$ increases in mesophyll cells.

Arabidopsis expressing GCaMP3 under the control of the RBCS1A promoter was exposed to *Z*-3-HAL that volatilized from a solution placed at a distance of 5 mm from the tip region.

Supplementary Movie 10. *Z*-3-HAL–induced $[Ca^{2+}]_{cyt}$ increases in vasculature cells.

Arabidopsis expressing GCaMP3 under the control of the SULTR2;2 promoter was exposed to *Z*-3-HAL that volatilized from a solution placed at a distance of 5 mm from the tip region.

Supplementary Movie 11. *Z*-3-HAL–induced $[Ca^{2+}]_{cyt}$ increases in epidermal cells.

Arabidopsis expressing GCaMP3 under the control of the ATML1 promoter was exposed to *Z*-3-HAL that volatilized from a solution placed at a distance of 5 mm from the tip region.

Supplementary Movie 12. High-resolution imaging of *Z*-3-HAL–induced $[Ca^{2+}]_{cyt}$ increases in guard cells.

Arabidopsis expressing GCaMP3 under the control of the GC1 promoter was exposed to *Z*-3-HAL, and Ca^{2+} signals at the cellular levels were observed using a confocal microscope.

Supplementary Movie 13. High-resolution imaging of *Z*-3-HAL–induced $[Ca^{2+}]_{cyt}$ increases in mesophyll cells.

Arabidopsis expressing GCaMP3 under the control of the RBCS1A promoter was exposed to *Z*-3-HAL, and Ca^{2+} signals at the cellular levels were observed using a confocal microscope.

Supplementary Movie 14. High-resolution imaging of *Z*-3-HAL–induced $[Ca^{2+}]_{cyt}$ increases in epidermal cells.

Arabidopsis expressing GCaMP3 under the control of the SULTR2;2 promoter was exposed to *Z*-3-HAL, and Ca^{2+} signals at the cellular levels were observed using a confocal microscope.

Supplementary Movie 15. High-resolution imaging of *Z*-3-HAL–induced $[Ca^{2+}]_{cyt}$ increases in *Arabidopsis* leaves expressing GCaMP3 driven by the 35S promoter.

Arabidopsis expressing GCaMP3 under the control of 35S promoter was exposed to *Z*-3-HAL, and Ca^{2+} signals at the cellular levels were observed using a confocal microscope.
